# “There is no free here, you have to pay”: actual and perceived costs as barriers to intermittent preventive treatment of malaria in pregnancy in Mali

**DOI:** 10.1186/s12936-016-1210-0

**Published:** 2016-03-12

**Authors:** Meredith C. Klein, Steven A. Harvey, Hawa Diarra, Emily A. Hurley, Namratha Rao, Samba Diop, Seydou Doumbia

**Affiliations:** Department of International Health, Johns Hopkins Bloomberg School of Public Health, 615 N. Wolfe Street, Baltimore, MD 21205 USA; Techniques and Technologies of Bamako, Faculty of Medicine and Odontostomatology, University of Sciences, Bamako, Mali

**Keywords:** Malaria, Malaria in pregnancy, Intermittent preventive treatment of malaria in pregnancy, Cost, Qualitative research, Mali, West Africa

## Abstract

**Background:**

“There is no free here,” the words of a Malian husband, illustrate how perceptions of cost can deter uptake of intermittent preventive treatment of malaria in pregnancy (IPTp). The Malian Ministry of Health (MOH) recommends a minimum of three doses of IPTp at monthly intervals. However, despite a national policy that IPTp be provided free of charge, only 35 % of pregnant women receive at least one dose and less than 20 % receive two or more doses.

**Methods:**

This study explored perceptions and experiences of IPTp cost in Mali and their impact on uptake, using qualitative interviews and focus groups with pregnant women, husbands and mothers-in-law. Study team members also interviewed and observed health workers at four health centres, two in Sikasso Region and two in Koulikoro.

**Results:**

Despite national-level policies, actual IPTp costs varied widely at study sites—between facilities, and visits. Pregnant women may pay for IPTp, receive it free, or both at different times. Health centres often charge a lump sum for antenatal care (ANC) visits that includes both free and fee-based drugs and services. This makes it difficult for women and families to distinguish between free services and those requiring payment. As a result, some forego free care that, because it is bundled with other fee-based services and medications, appears not to be free. Varying costs also complicate household budgeting for health care, particularly as women often rely on their husbands for money. Finally, while health facilities operating under the cost-recovery model strive to provide free IPTp, their own financial constraints often make this impossible.

**Conclusions:**

Both actual and perceived costs are currently barriers to IPTp uptake. Given the confusion around cost of services in the two study regions, more detailed national-level studies of both perceived and actual costs could help inform policy and programme decisions promoting IPTp. These studies should evaluate both quantitatively and qualitatively the cost information provided to and understood by pregnant women and their families. Meanwhile, unbundling free and fee-based services, making clear that IPTp is free, and ensuring that it is provided at no cost could help increase uptake. Free community-based distribution might be another route to increased uptake and adherence.

## Background

“There is no free here,” said a Malian husband during a focus group in rural Baguinéda village, “you [always] have to pay.” The comment-in response to a question about antenatal care—highlights one key barrier to the uptake of intermittent preventive treatment of malaria in pregnancy (IPTp). Officially in Mali, IPTp is provided at no cost to the patient. However, inconsistently applied policies and a systemic need for revenue generation lead to hidden charges and both provider and patient confusion about precisely what is and what is not free. Many pregnant women forego IPTp as a result.

Malaria in pregnancy can lead to premature birth, low birth weight, and maternal and neonatal anaemia, all conditions associated with increased risk of spontaneous abortion, stillbirth, and neonatal and maternal death [1, 2]. Given these negative effects, international agencies led by the World Health Organization (WHO) have developed a three-pronged approach to prevention using (1) sulfadoxine-pyrimethamine (SP) for IPTp; (2) insecticide-treated bed nets (ITNs); and (3) rapid diagnosis and treatment of malaria. Current international guidelines call for pregnant women to receive IPTp doses at least 1 month apart at their antenatal care visits from the start of their second trimester through delivery and ideally under directly observed therapy [3]. These guidelines also recommend at least four antenatal care visits over the course of a pregnancy [3]. As of 2013, the year data collection for this study began, the Malian Ministry of Health (MOH) guidelines were revised in line with the WHO recommendation and stipulate at least three doses of IPTp at monthly intervals by directly observed therapy beginning in the second trimester [4]. Despite this national policy, only 35 % of pregnant women receive at least one dose of IPTp and less than 20 % receive two or more doses [5]. Although Malian policy dictates that all pregnant women should receive IPTp and other preventive services free of charge, cost remains a barrier to both antenatal care (ANC) and IPTp [2, 4].

The cost structure of the Malian health system has evolved over recent decades. Beginning in 1989, Mali decentralized health services to the community level in line with the Bamako Initiative promulgated jointly by UNICEF and WHO [6]. Announced in 1987, the Bamako Initiative advocated for decentralization and cost-recovery as a means of improving health facilities and services and standardizing quality of care [7]. In Mali, the Bamako Initiative took the form of locally managed health facilities, governed by Community Health Associations (ASACOs) [6, 8]. Supplementing low government investment, the ASACO implemented a cost-recovery model based on user fees to generate operational revenue. Through this model of community financing, the Bamako Initiative aimed to expand access to basic health services and ensure equity in the provision of care. Over time, however, the policy had the opposite effect: Many of the most vulnerable were unable to pay user fees and thus unable to access services [8]. Recognition of this problem led to a global health strategy reversal abolishing user fees in the early 2000s [9]. Although at the global level the Bamako Initiative was ultimately abandoned, in Mali the decentralized ASACO model and health facility dependence on cost-recovery continue. Since health centres must still generate revenue to cover their operating budgets, providing free medication (a major revenue source) is at the uncomfortable intersection of central-level policy and peripheral-level operational reality. This study examines how both actual and perceived costs serve as barriers to IPTp uptake among women in Mali within this post-Bamako Initiative context.

## Methods

### Study area and population

This study took place August 2013–April 2014 in four rural health zones, two in Sikasso and two in Koulikoro. These are the two most populous of Mali’s eight regions, with 2.6 and 2.4 million inhabitants, respectively [10]. The vast majority of inhabitants live in rural areas: 84 % in Sikasso, 95 % in Koulikoro. In both regions, malaria is endemic, and SP uptake comparable to the national and rural figures (Table 1) [5]. Each health zone encompasses one or more rural villages served by a common community health centre (*Centre de Santé Communautaire*, or CSCOM). Additional data were collected in 2015 from key informants at the district and regional levels of both the National Malaria Control Programme and the Reproductive Health Programme.

**Table 1:** Percentage of pregnant women attending ANC and receiving SP during pregnancy in Mali [5]

	Total (national)	Sikasso region	Koulikoro region
% attending at least 1 ANC visit	74.1		
% receiving SP during an ANC visit	34.6	32.4	29.3
% attending 2+ ANC visits	69.2		
% receiving 2+ doses SP during ANC visits	19.9	21.0	16.4

### Study design, sampling and data collection

The overall study objective was to identify principal barriers to the uptake of at least two doses of IPTp among pregnant women in Mali. To assess these barriers, the study used qualitative methods in rural villages within each health zone. These included: semi-structured in-depth interviews (IDIs), and focus-group discussions (FDGs), ANC observations and record reviews at CSCOMs, and key informant interviews (KIIs) at district and regional facilities.

Within each health zone, the study team used purposive sampling to recruit participants with a range of perspectives on IPTp. Purposive sampling in this context refers to purposively selecting categories of informants likely to have knowledge about or influence upon pregnant women’s uptake of IPTp. Table 2 lists the categories of participants enrolled in the study and the number of participants per category. Within each category, individual participants were identified by a combination of convenience and snowball sampling [11].

**Table 2:** Data collection by region and type of participant

	Koulikoro region	Sikasso region	Total
*In-depth interviews (IDIs)*	*15*	*22*	*37*
Pregnant or breastfeeding woman	6	9	15
Midwife or auxiliary nurse	2	2	4
Pharmacist	2	1	3
Physician/CSCOM head	–	4	4
ASACO president	1	–	1
Community health worker	1	1	2
NGO worker	–	1	1
District official (KII)	3	3	6
Mayor	–	1	1
*Focus group discussions (FGDs)*	*12*	*14*	*26*
Elderly women	3	5	8
Teachers	1	2	3
Community health workers	2	3	5
Husbands	3	2	5
Village officials	1	1	2
Pregnant or breastfeeding women	2	–	2
ASACO	–	1	1
*Observations*			
ANC visit	10	10	20

The data collection team included three physicians and one sociologist associated with the School of Public Health at the University of Sciences, Techniques, and Technologies of Bamako plus one doctoral student from the Johns Hopkins University Bloomberg School of Public Health (author EH) who is fluent in Bambara. Two of the University of Bamako team members had extensive experience with qualitative interviewing; the other four had more limited experience. Prior to beginning data collection, University of Bamako team members underwent 1 week of training in qualitative methods and study objectives provided by the principal investigator (SAH) and the Hopkins doctoral student. All data collectors were female.

IDIs and FGDs were audio-recorded and all participants provided oral consent. Interviewers read the oral consent form to each participant. In conformity with IRB guidelines, the interviewer then signed her own name on the form confirming that the participant had consented to participation. Interviewers used funnelling, a questioning technique that begins with broader questions in order to examine whether and how a participant spontaneously raises a specific topic of interest (in this case, IPTp) [12]. Participants were asked broader questions to elicit schemas of (a) health and illness during pregnancy, (b) disease prevention and medications during pregnancy, and (c) experiences with ANC. Later, participants were probed about more specific topics related to malaria, IPTp, SP and long-lasting insecticidal nets (LLINs). During key informant interviews, data collectors asked district and regional health officials about content and implementation of IPTp policy and the influence of community and health systems on IPTp uptake. IDIs and FGDs were conducted in Bambara, with the exception of some key informants and clinicians who preferred French.

The study team recruited a convenience sample of five women per CSCOM for ANC observations. Each participant provided verbal informed consent for the data collector to accompany her and observe her entire visit. The data collector completed a structured observation form covering the patient-provider interaction, clinical exam, content of patient counselling, LLIN distribution if it occurred, and prescription, acquisition, and administration of pharmaceuticals. When participants were referred to the health facility pharmacy to fill prescriptions or retrieve medication, a data collector shadowed the process, confirming paid or free provision of IPTp by asking the woman and/or verifying details on the prescription receipt. Data collectors also took unstructured notes detailing the appointment. In addition, the team reviewed maternity and pharmacy record books at each CSCOM for SP stock and distribution information.

### Data analysis

Data analysis was an iterative, collaborative process. Interviewers wrote reflective notes following each data collection session and discussed developing themes at regular debriefing meetings. The sampling strategy and interview guides were revised as new questions emerged.

Audio recordings of IDIs, FGDs, and KIIs were transcribed into the language in which they were conducted. Transcripts in Bambara were subsequently translated into French. Primary documents (transcripts and observation notes) were uploaded into ATLAS.ti (version 7) for topical coding [13]. The research team began coding inductively and periodically revised the codebook as new themes emerged. Data were interpreted through continued memo writing and debriefing during and beyond the coding process [14].

### Ethical considerations

Ethical approval was obtained by the Institutional Review Boards of the Johns Hopkins Bloomberg School of Public Health (#4848) and the University of Bamako: Faculty of Medicine, Pharmacy, and Odontology. Prior to data collection, researchers obtained permission from village, CSCOM and CSREF leaders as well as informed consent from participants.

## Results

The study team completed 37 IDIs, 26 FGDs, and 20 observations. Table 2 shows the breakdown by region and type of participant for each method.

### Actual and perceived cost as an obstacle to IPTp

Despite Mali’s policy of providing IPTp free to all pregnant women, interviews with both women and health care providers demonstrate that costs vary widely. In focus group discussions, pregnant women would often debate about fees and what they were for, citing different experiences even at the same health facility. This variability even within a single FGD indicated that cost variability impacted all women seeking IPTp irrespective of demographic characteristics. While some women reported that they did receive free SP, many reported that SP was included in a prescription they would have to pay to fill at the health centre pharmacy. Women often did not know the costs of individual medications included in prescriptions since the amounts were presented as a lump sum at the pharmacy counter as found in both interview and focus group settings and in structured observations.

*If you buy the medications one by one you can find out their price, but they write a prescription and send you to the health centre pharmacy to buy them where they total the price of all the medications. If you have the money you buy the medications. Otherwise, you just buy the priority ones and afterwards you can come back and buy the rest […] Even if you can’t find drugs for free, if it’s less expensive that would make things easier.* (30-year-old pregnant woman, Koulikoro)

Lump sum totals make it difficult for women and their families to decipher which medicines are free and which require payments. To determine individual prices requires additional probing and negotiation between the payer and the pharmacist. Patients may see more familiar medications such as vitamins and iron as a priority while leaving behind lesser-known medications like SP. Nearly all study participants, both male and female, identified malaria as a major risk to pregnant women, but only a minority spontaneously identified SP as a drug given to prevent malaria in pregnancy. In fact, many interviewees mentioned that they wished there were a pill pregnant women could take to protect them against malaria.

Aware that financial concerns may prevent some women from filling ANC prescriptions, some providers try to explain that there is no cost for IPTp. As a 32 year-old Koulikoro midwife explained:

*When we prescribe SP, we tell them to go pick it up at the pharmacy… But once they have the prescription they just go home saying they don’t have the money. We tell them, “even if you don’t have the money, go to the pharmacy. There is a free prescription there that they will give you.” Despite that many of them don’t go to the pharmacy and get it.*

However, ANC observations revealed that midwives and pharmacists were inconsistent about explaining to patients that IPTp was free, so it is unclear what proportion of women understood this. The majority of pregnant and recently-pregnant respondents reported experiences of both paid and free IPTp administration over time. Initial doses (usually the first and sometimes the second) might be provided at no charge during ANC visits, with additional treatments provided for a fee. All cost assumptions and experiences change, however, in the case of stock-outs. During stock-outs, women receive a prescription they must pay to fill at either the health centre pharmacy or a separate private pharmacy, even for initial doses. Health providers report that while SP stock-outs are not a persistent challenge, they do occur and can last several months.

### Actual costs as an obstacle to attending ANC

Despite the inconsistencies of IPTp and general pharmaceutical costs, a known and expected expense associated with pregnancy at health facilities is the purchase of an ANC card. At the first ANC visit, a pregnant woman pays for her card. The card is then used to track visits and health information during the pregnancy. At the time of delivery, additional fees are assessed. As noted during observations, however, women may incur additional costs beyond the ANC card purchase during follow-up visits. In some facilities women are expected to pay a small fee at each consultation visit to cover the price of gloves or other routine materials needed by the health care providers. Additional expenses may be a separate lump sum paid during initial ANC visits or periodic amounts collected at the start of each visit. Such variable fees are often tied to cost recovery efforts to support the ongoing operation of the health facility.

ANC card fees, other incidental charges, and delivery costs deter women from attending regular ANC visits. The previously quoted midwife from Koulikoro summarized the situation:

*It’s a money problem because even when pregnant women come for their first ANC visit, if we calculate out the total including ANC and delivery fees and it goes too high they don’t pay at all and they don’t come back again.*

When explaining ANC, health workers tend to mention the costs associated with the services rather than the elements that are free. As a result, some women forego both free and fee-based care. Nationally, 75 % of pregnant women attend at least one ANC visit but only 55 % ultimately deliver at a health facility [5]. Costs may account for a portion of this discrepancy.

### Financial decision-making power within households

Across all study sites, interviewees cite family patriarchs as the decision-makers about ANC attendance. Community members, including pregnant women, state that permission to attend ANC appointments and money to finance the visits comes from the pregnant woman’s husband, his older brother, or his father. “*Once your wife gets pregnant*, explained one Sikasso husband, *“you have to prepare for all the expenses*.” But “prepar[ing] for all the expenses” does not necessarily indicate that a husband has agreed in advance to incur them. A woman may have to negotiate with her husband or another male relative about each ANC visit.

In providing financial support, some men accompany their wives to the health facility and handle financial transactions directly. This can create an important disconnect between costs and the services with which they are associated as the men almost never attend the counselling sessions, but simply pay at the pharmacy once the visit ends. The majority of women enter counselling sessions alone even if accompanied by their husband to the facility. Men have little interaction with the health workers during their wives’ pregnancies and often do not know the specifics of the counselling, guidance, or medications provided. Although the study did not elicit specific reasons for husbands’ lack of involvement, ANC consultations in Mali are generally considered the purview of women—a setting where male participation would seem unusual. Among the men who do not accompany their wives, many take their wives’ prescriptions from the visit and return to the pharmacy on their own to pay any fees directly. This finding reinforces observations that women often do not know what costs are incurred for the services they receive because, *“it’s our husbands that pay, so we don’t know the price”* (35-year-old nursing mother in Sikasso). Additionally the husbands themselves are not cognizant of what amounts they are paying for which medicines. As one husband in Sikasso explained, *“If you’re educated you can figure out the prices on the prescription. They typically just prescribe everything together and don’t tell you the different prices or products.”*

Costs can also create tension within families. One observer noted that a husband and wife were visibly stressed when filling the prescriptions at the pharmacy—worried about the number of medications needed and the cost to procure them all. This tension can extend beyond the health facility, particularly when women are sent to ANC visits on their own. The experience of a 40 year-old Sikasso mother illustrates the issue:

*“At my first and second ANC visits I had to pay by other means. When I asked my husband to reimburse me, he refused […] another time I had to send my daughter to him to ask for money when I was in the hospital and he said he didn’t have any so I ended up borrowing 5,000 CFA [US2015 $8.37] from my mother. Then we had an argument.*

The variability of health centre costs combined with the uncertainty of financial support from their husbands poses an additional obstacle for pregnant women seeking regular ANC. Beyond their husbands, women often cited mothers-in-law as arbiters of financial decisions related to pregnancy. Women only cited themselves when probed further about permission and support in the absence of their husbands or mothers-in-law. Despite this apparent lack of autonomy, women reported that ultimately it was up to them to find the resources to pay for their own care—particularly in asking other family members for money. A focus group discussion exchange from Koulikoro illustrates the situation:

*Pregnant woman 1: Lots of husbands don’t give money [for ANC]*

*Pregnant woman 2: Many of the men do not give money [for ANC]. We have to find ways for ourselves to get treatments by what few means we have.*

*Pregnant woman 3: Yes, husbands will just tell you to go take traditional medicines.*

The difficulty women face in finding alternative sources of money to pay for ANC may reduce the frequency and level of services that they are able to obtain.

### Health facility challenges to providing free SP

Health facilities acknowledge that their efforts to provide free SP in line with national policy sometimes fall short. “*It could be that today we don’t have the means*,” commented one ASACO President in Koulikoro. “*We like to be able to provide [IPTp] for free, but it depends on the financial circumstances at the CSCOM*.” Since health facilities must continue to generate revenue to maintain core operations, their ability to provide free IPTp varies over time. Ultimately the pricing scheme is left to the discretion of the ASACO, and practices differ by community. Within a given ASACO, a financial shortfall could lead to fee increases even for medicines or services that are supposed to be free. “*Free provision is a challenge*,” explained one reproductive health manager in Sikasso, “*its timeliness, permanence and continuity are a real problem*.” This sort of unpredictability can deter women from continuing their IPTp regimen if only their first dose is free, or prevent them from taking IPTp at all if the costs are too high. “*Once [*pregnant women*] have benefited from free IPTp*,” noted one doctor at a referral health centre in Sikasso, *“they do not take well to paying for it.*”

In addition to stocking free SP for IPTp, some health facilities also sell it for curative treatment contrary to both national policy and international recommendations. Apart from going against WHO guidelines for the strict use of SP only for IPTp and not as a curative treatment, this practice also further contributes to cost complication. As noted during observations, women can be mistakenly charged for SP doses if the prescription is not explicit or the pharmacist does not know that the dosage is intended as a free course of IPTp. Observers also documented different understandings about IPTp provision between clinical and pharmacy personnel. Within one health facility, for example, the pharmacy staff stated that both the first and second doses of IPTp were provided free while clinicians indicated that only the first dose was free.

## Discussion

**Fig. 1:**
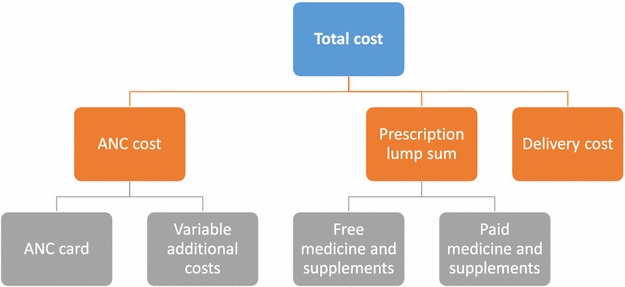
Illustrative facility-based pregnancy care cost components. Total costs incurred within a health facility during pregnancy can be broken down into components shown above. While IPTp costs may be included in prescriptions as either a free or paid treatment, this figure highlights the varying levels of expenses just within the facility which contributes to the differing perceptions and confusion around costs

Findings from this study show three key ways in which costs inhibit IPTp uptake. First, although national policy dictates free provision, pregnant women find themselves obligated to pay for SP when health facilities provide a first dose free but charge for subsequent doses, when stock-outs occur, and when pharmacies charge for a preventive dose as if it were curative. These inconsistencies may discourage women from pursuing IPTp because they see it as a service that requires payment even in cases when it does not. The fact that different health workers have different understandings about the policy further confounds the issue. At a single facility, one health worker may tell a pregnant woman that IPTp is free while another tells her there is a charge, further contributing to the overall impression that “*there is no free here, you [always] have to pay*.” Second, pregnant women often lack independent access to funds and thus rely on a husband, older brother-in-law, or other family member to pay any ANC-related expenses. Since this other family member is unlikely to have attended the ANC consult, he or she is more likely to know the price of a given service or medication than to understand its value. This may make him or her less inclined to see the expense as justified. Third, costs related to pregnancy and childbirth may discourage participation even in the free aspects of ANC. While most ANC services are free, pregnant women must purchase an ANC card at the initial visit and pay a fee for in-facility delivery. In between are hidden or incidental charges for routine supplies such as gloves that the CSCOM operating budget may be unable to cover. Prescription bundling adds to the confusion: When a pregnant woman arrives at the CSCOM pharmacy with a list of medications and the pharmacy attendant quotes a single price for the entire list, she and/or her husband or other family member may perceive that they are paying for IPTp when the charges are for other medications. Malians continue to pay out of pocket for 50 % of all health expenditures, and such costs remain important barriers to access [6]. This finding is consistent with the conclusions of a recent single-community pilot programme in Mali that providing free drugs is not enough to ensure high uptake when user fees remain in place [21]. Despite WHO recommendations that IPTp be provided by directly observed therapy, the observations and accounts of patients filling pharmacy prescriptions to obtain SP demonstrate that this is not consistently practiced in Mali, an issue addressed elsewhere (Hurley et al, pers. comm.). Though indirect and opportunity costs associated with attending clinic visits were not included in our study, they are also well documented as major barriers to care [15–17]. Figure 1 details components of cost for facility-based pregnancy care.

It is tempting to recommend elimination of all user fees related to pregnancy and childbirth as the most straightforward solution. However, such a recommendation would only be feasible if the central health system is willing and able to assume the additional costs. CSCOMs and CSREFs on their own could not cover these costs under the current funding model. Although the Bamako Initiative has been abandoned in favour of what are supposed to be more equitable health care provision practices, ASACOs and the CSCOMs that they manage continue to depend on locally generated revenue. Chronically underfunded, CSCOMs depend upon user fees and pharmaceutical sales to remain viable [9].

To eliminate user fees as an obstacle to IPTp uptake, alternate administration methods may be preferable to the current facility-based practices. While the present study focuses exclusively on facility-level costs associated with IPTp, there are numerous external costs that prevent IPTp uptake. Transportation costs and the opportunity costs of taking time off to visit health centres play a significant role in health care access. Direct distribution at the community level would, however, largely eliminate these external costs as a barrier. Several studies in sub-Saharan Africa have found that having community health workers distribute free SP directly to women significantly increased uptake [18–20]. While IPTp was offered free of charge both in the facilities and by community health workers, the direct provision of service to the women at their homes or in their communities had a significant impact. Although these studies have shown differing impacts on ANC attendance, each has shown that community-based distribution of SP greatly increases IPTp uptake.

## Limitations

In the present study, geographic scope was limited to only two of Mali’s eight regions. The sample was small, purposive, and limited to four CSCOMs and their catchment areas. Statistically, study results are not generalizable beyond the study areas. Nevertheless, though the sample was limited and the study team did not collect quantitative cost data on IPTp, general findings are in line with others studies in sub-Saharan African on the effect of cost on care-seeking behaviours [15–17, 21]. Given that government subsidies are lowest at the peripheral CSCOM level, the cost-recovery burden for these facilities is much higher than for referral hospitals and regional facilities. Cost recovery does remain an issue throughout the system, however, and with decentralized services the experiences at the CSCOM level represent a crucial touch point between the health system and population.

While all interviewers were female, data from both female and male FGDs and IDIs were consistent. The importance of cost as a barrier to IPTp in Mali is evidenced by how frequently it emerged in interviews with pregnant women, family members, and health care providers. Additionally, although data from observations indicated consistent practices within facilities, these were conducted over a 1–2 day period at each of the four included facilities. Given the variance in cost experiences reported by women in FGDs and IDIs, additional longitudinal observations would likely reinforce these qualitative findings.

## Conclusions and recommendations

Both actual and perceived costs are currently barriers to IPTp uptake in Mali. The continuing importance of cost-recovery schemes at the community level hamper policy efforts to eliminate user fees for priority services. Dependent on community financing, community health centres (CSCOM) are unable to abolish user fees, relying on them for operational revenue.

Given the confusion around cost of services in the two study regions, more detailed national-level studies of both perceived and actual costs could help inform policy and programme decisions promoting IPTp. These studies should evaluate both quantitatively and qualitatively the cost information provided by health facilities and pharmacies to pregnant women and their families. Facility-level operational research might also unveil innovative ways to provide free services and drugs within the existing cost-recovery system, or identify more effective and efficient health financing structures. Free community-based distribution of SP via health workers would be another route for increasing IPTp uptake and adherence, as demonstrated in the aforementioned studies [18–20]. Such a policy could be particularly useful given the WHO guidance supporting monthly IPTp administration by directly observed therapy.

## References

[1] WHO. A strategic framework for malaria prevention and control during pregnancy in the Africa region. Brazzaville: World Health Organization Regional Office for Africa; 2004.

[2] Gomez PP, Gutman J, Roman E, Dickerson A, Andre ZH, Youll S (2014). Assessment of the consistency of national-level policies and guidelines for malaria in pregnancy in five African countries. Malar J.

[3] WHO. Updated WHO Policy Recommendation (October 2012). Intermittent preventive treatment of malaria in pregnancy using sulfadoxine-pyrimethamine (IPTp-SP). Geneva: World Health Organization; 2012.

[4] Ministère de la Santé (2013). Programme national de lutte contre le paludisme: politique national de lutte contre le paludisme.

[5] INFO-STAT, Cellule de Planification et de Statistiques CPS, Institute National de la Statistique INSTAT, ICF International. Enquête Démographique et de Santé EDSM-V 2012–2013. Bamako; 2013.

[6] Lamiaux M, Rouzaud F, Woods W (2011). Private health sector assessment in Mali: the post-Bamako Initiative reality.

[7] Nolan B, Turbat V (1995). Cost recovery in public health services in Sub-Saharan Africa.

[8] Ridde V (2003). Fees-for-services, cost recovery, and equity in a district of Burkina Faso operating the Bamako initiative. Bull World Health Organ.

[9] Ridde V (2011). Is the Bamako initiative still relevant for West African health systems?. Int J Health Serv.

[10] Ministère de l’economie et des finances: Quatrième recensement général de la population et de l’habitat, 2009: Résultats définitifs. Bamako; 2011.

[11] Yin RK (2016). Qualitative research from start to finish.

[12] Morgan DL. Focus groups as qualitative research. Thousand Oaks: SAGE Publications; 1997.

[13] ATLAS.ti Scientific Software Development GmgH: ATLAS.ti. 7.5.6 2015.

[14] Charmaz K. constructing grounded theory, 2nd edn. Thousand Oaks: SAGE Publications; 2014.

[15] Pell C, Straus L, Andrew EVW, Meñaca A, Pool R (2011). Social and cultural factors affecting uptake of interventions for malaria in pregnancy in Africa: a systematic review of the qualitative research. PLoS One.

[16] Mubyazi GM, Bloch P, Magnussen P, Olsen ØE, Byskov J, Hansen KS (2010). Women’s experiences and views about costs of seeking malaria chemoprevention and other antenatal services: a qualitative study from two districts in rural Tanzania. Malar J.

[17] Hill J, Hoyt J, van Eijk AM, D’Mello-Guyett L, Ter Kuile FO, Steketee R (2013). Factors affecting the delivery, access, and use of interventions to prevent malaria in pregnancy in sub-Saharan Africa: a systematic review and meta-analysis. PLoS Med.

[18] Mbonye AK, Bygbjerg IC, Magnussen P (2007). A community-based delivery system of intermittent preventive treatment of malaria in pregnancy and its effect on use of essential maternity care at health units in Uganda. Trans R Soc Trop Med Hyg.

[19] Msyamboza KP, Savage EJ, Kazembe PN, Gies S, Kalanda G, D’Alessandro U (2009). Community-based distribution of sulfadoxine-pyrimethamine for intermittent preventive treatment of malaria during pregnancy improved coverage but reduced antenatal attendance in southern Malawi. Trop Med Int Health.

[20] Okeibunor JC, Orji BC, Brieger W, Ishola G, Otolorin E, Rawlins B (2011). Preventing malaria in pregnancy through community-directed interventions: evidence from Akwa Ibom State, Nigeria. Malar J..

[21] Ponsar F, Van Herp M, Zachariah R, Gerard S, Philips M, Jouquet G (2011). Abolishing user fees for children and pregnant women trebled uptake of malaria-related interventions in Kangaba, Mali. Health Policy Plan.

